# Effect of environmental variables on mercury accumulation in sediments of an anthropogenically impacted tropical estuary (Buenaventura Bay, Colombian Pacific)

**DOI:** 10.1007/s10661-023-11721-9

**Published:** 2023-10-14

**Authors:** Andrés Molina, Guillermo Duque, Pilar Cogua

**Affiliations:** 1 Grupo de investigación en Ecología y Contaminación Acuática, Universidad Nacional de Colombia, Sede Palmira, Palmira, Colombia; 2 Universidad Nacional de Colombia, Sede Palmira, Facultad de Ingeniería y Administración, Palmira, Colombia; 3 Universidad de Santiago de Cali, Facultad de Ciencias Básicas, Cali, Colombia

**Keywords:** Total Mercury, Sediments, Organic Matter, Salinity, GAM, Buenaventura Bay

## Abstract

Estuaries are the main entry areas of mercury to the marine environment and are important to understand the effect of this contaminant on marine organisms, since it accumulates in the sediments becoming available to enter the food trophic chain. This study aims to determine the environmental variables that mainly influence the spatiotemporal dynamics of total mercury accumulation in sediments of tropical estuaries. Sediment samples were collected from interior and exterior areas of the estuary during the dry and rainy seasons, representing the spatiotemporal gradients of the estuary. The grain size, organic matter content (OM), and total mercury concentration (THg) of the sediment samples were determined. In addition, salinity, temperature, dissolved oxygen, and pH of the water column associated with each sediment sample were assessed. The variations in environmental conditions, OM and THg in sediment were in accordance with a gradient which goes from conditions influenced by fresh water in the inner estuary to conditions influenced by sea water in the outer part of the estuary. The OM and THg in sediments presented similar variation patterns; they were higher in the rainy season than in the dry season and in the interior area of the estuary than in the exterior area. Despite the complex dynamic observed in the distribution and accumulation processes of mercury in sediments, these processes could be modeled from OM and salinity parameters. Due to the correlations found, in the process of accumulation of mercury in sediments the OM could represents the pathway of transport and accumulation of THg, and salinity could represent the influence of the hydroclimatic variations and environmental gradients of the estuary.

**Supplementary Information:**

The online version contains supplementary material available at 10.1007/s10661-023-11721-9.

## Introduction

Estuaries represent the transition from a freshwater system to a marine environment, gathering the advantages and disadvantages of both ecosystems (Cooper et al., 1994; Elliott & Quintino, 2007). The environmental dynamic of estuaries facilitates metal speciation and mercury methylation, which makes estuaries an important ecosystem to study mercury pollution (Morel et al., 1998; Rodríguez Martín-Doimeadios et al., 2004). Mercury is a metallic element that can be naturally found in ecosystems in different chemistry forms (Morel et al., 1998; Olivero & Johnson, 2002), and can be released to the environment through natural processes, including volcanic eruptions and soil erosion, and through anthropic activities, such as fossil fuel burning and gold mining (Weinberg, 2010). Moreover, it is transported long distances through the atmosphere where it can be attached to the suspended matter and then deposit in terrestrial and aquatic ecosystems, affecting organisms including humans (Mason & Fitzgerald, 1993; Tsutsumi et al., 2019; UNEP, 2019).

Mercury effects have a high environmental impact, altering the ecosystem balance and human health (Pereira et al., 2010). Its effect on ecosystems varies depending on whether its inorganic or organic forms are more available (Engstrom, 2007; Harris et al., 2003; Walker, 2008), being the organic mercury form the most dangerous, due to its toxicity and lipophilicity (Tavares et al., 2011). This characteristic facilitates the bioaccumulation and biomagnification of mercury, being able to reach higher concentrations in organisms than in the environment, and at high trophic levels (Bisi et al., 2012; Qu et al., 2022; Trevizani et al., 2021).

After its incorporation in the estuaries through direct anthropic release, atmospheric deposition, and river runoff, mercury leads to accumulation processes in biotic and abiotic compartments as well as active flow processes in the ecosystem (AMAP/UNEP, 2015; Jaingam, 2018; Tsutumi et al., 2019). The organic matter (OM) content is a key factor in the flux and accumulation of mercury in sediments, since it can bind to the sediment by forming covalent bonds due to its affinity for sulfur-containing functional groups found in the organic molecules of OM, thus allowing their mobility, speciation, and controlling their bioavailability (Han et al., 2006; Lacerda & Fitzgerald, 2001; Schuster, 1991). Benthic organisms are crucial in these ecosystems, as they participate in the processes of organic matter exchange in the water–sediment interface through bioturbation and feeding processes, contributing to the active flow and accumulation of various metals in estuaries, including mercury (Cardoso et al., 2008; Jędruch & Bełdowska, 2020; Jędruch et al., 2019; Jonsson et al., 2022).

Marine sediments function as temporary deposits of mercury, where the metal is mostly associated with the fine-grain fraction and OM content from various origins (Azaroff et al., 2019). The OM from external sources and also that derived from dead plankton and excretions adhere to the degraded detritus deposits in the sediments, while the non-degradable fractions, such as mercury, tend to accumulate (Tsutsumi et al., 2019). In the benthic environment, the invertebrates accumulate mercury by food and organic matter, through which it becomes available to the benthic fishes (Jaingam, 2018). Due to this process, accelerated pathways of mercury accumulation have been detected in benthic environments, which are important to understand mercury dynamics in estuaries (Jaingam, 2018; Tsutsumi et al., 2019). Physicochemical variables, types of sediments, sediments resuspension, currents, seasonal factors, and the structure of communities, are among the elements influencing mercury dynamics in coastal ecosystems (Acquavita et al., 2012; Bratkič et al., 2013; Costa et al., 2012; Fox et al., 2014; Kim et al., 2004; Taylor et al., 2012). Rainfalls are a determinant factor in mercury dynamics of tropical areas as they are associated with the runoff and increase in flow of rivers as they are the principal sources of mercury in the aquatic ecosystems (Barletta et al., 2012).

Mercury accumulation in sediments has been reported in several estuaries worldwide, and complex spatial and temporal processes associated with freshwater flows and physicochemical characteristics of sediments have been identified (Cukrov et al., 2020; Garcia-Ordiales et al., 2018; Kehrig et al., 2003; Quintana et al., 2020; Vane et al., 2020; Zhao et al., 2019). Moreover, active mercury dynamics have been observed in estuaries from South America (Barletta & Lima, 2019; Costa et al., 2012) including Colombia (Alonso et al., 2000; Burgos et al., 2014; Campos et al., 2015; Cogua et al., 2012). The literature regarding mercury presence in Buenaventura Bay is scarce; however, mercury concentration is known to be increasing at an accelerated rate, and it could be dangerous for the coastal communities at the detected concentrations in estuarine fauna (Gamboa-García, 2017; Gamboa-García et al., 2020; Panesso, 2017). In this sense, between 1995 and 2002, the concentration of mercury in the water of Buenaventura Bay increased 22 times (Ospina & Peña, 2003), possibly associated with illegal gold mining (Le Billon et al., 2020). The total mercury concentration (THg) registered in sediments range from 0.2 to 0.6 µg/g (Velásquez & Cortés, 1997), similar to the values detected in other estuaries with an active mercury flow, such as Cartagena Bay (Colombian Caribbean) with 0.18 µg/g of THg in sediments (Cogua et al., 2012). In Buenaventura Bay, an increase of abnormalities in the erythrocytes nucleus were detected in fish: the flathead grey mullet (*Mugil cephalus*) and the golden mojarra (*Diapterus aureoles*) (Duque & Cogua, 2016), suggesting an effect on the communities of organisms that inhabit the Buenaventura Bay estuary (Martinez et al., 2019; Gamboa-García et al., 2020; Molina et al., 2020). Considering this context, the objective of this research was to determine the environmental variables (physicochemical variables of water, granulometry, and organic matter) that most influence the accumulation of total mercury in sediments of Buenaventura Bay, Colombian Pacific, an anthropogenically impacted tropical estuary.

## Methodology

### Site description

**Fig. 1:**
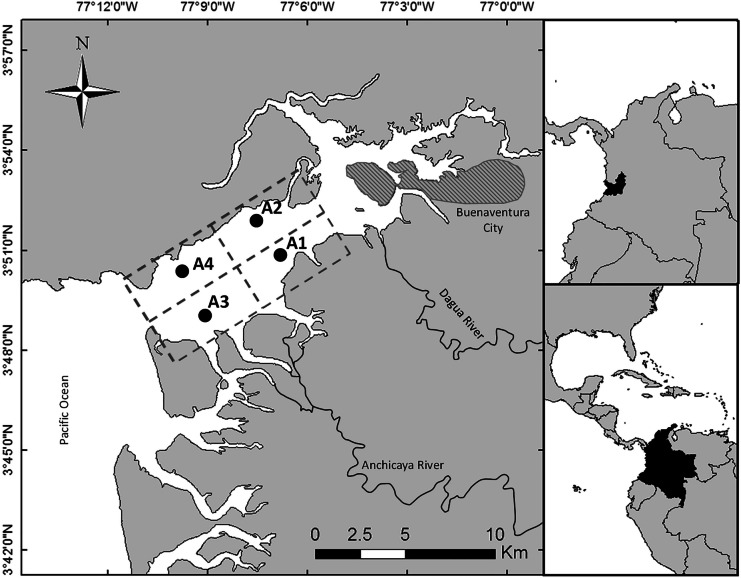
Estuary of Buenaventura Bay and the four sampling areas (A1, A2, A3, and A4). The urban area is marked with diagonal lines

The area of Buenaventura Bay is one of the most humid regions in the world and it is located in the Tropical Eastern Pacific (3°48ʹ09.99ʺ–3°52ʹ38.57ʺ N; 77°06ʹ30.75ʺ–77°09ʹ25.96ʺ W) (Fig. 1), and is situated in the Intertropical Convergence Zone and close to the Andes Mountains (Cantera & Blanco, 2001). This bay presents approximately 228 to 298 rainy days per year, an average of an annual rainfall of 6508 mm, a relative humidity between 80%–95% and a mean temperature of 25.9 °C (Lobo-Guerrero, 1993). In the dry season, from January to June, the average monthly rainfall range is 200–500 mm, and in the rainy season, from July to December, the average monthly rainfall ranges from 500 to >700 mm (Fig. A1a). The average depth of the estuary is 5 m and it has two main tributary rivers, the Anchicayá and Dagua rivers (427 m^3^ s^−1^), which can be catalogued as a positive estuary (Gamboa-García et al., 2018). This estuary can be considered a well-mixed system because the differences in salinity between the bottom and surface are lower than 2 (Otero, 2005).

In this study, Buenaventura Bay was divided into four areas depending on salinity gradient, geomorphology, and environmental characteristics (Fig. 1). In the interior part of the bay, the area closer to the urban zone with domestic and industrial wastewater discharges and effluents from illegal mining in the tributary rivers (potential sources of mercury of natural and anthropogenic origin), two areas were defined: Area 1 (A1) in the south with direct influence from rivers and Area 2 (A2) in the north with less influence from the rivers. In the exterior part of the estuary (the area with mainly marine influence), two other areas were defined: Area 3 (A3) in the south with some influence from the rivers and Area 4 (A4) in the north with principal influence from the sea. The influence of the rivers was determined by changes in salinity since there are no reliable updated studies on water flows and sedimentation in the bay. This ecosystem is highly intervened, harboring a population of 300,000 inhabitants in the proximities to the estuary (DANE, 2019), and is the most important port in Colombia (Diaz, 2007).

### Sampling method

Sediment samples were collected from a depth of 2.1 ± 0.6 m (mean ± standard deviation (SD)), and the physicochemical variables of the four areas of the estuary (Fig. 1) were determined in situ. Samples were collected four times in a year, in the months of April, June, September, and November of 2015. The samples of April and June were analyzed together as dry season and those of September and December as rainy season, following the analysis of the historic series of rainfalls (Fig. A1b). In each field trip, three replicates (one core each) were taken in each of the four sampling areas.

The environmental variables such as salinity, temperature (°C), dissolved oxygen (mg l^−1^), and pH were measured at the depth corresponding to the middle of the water column (Thermo Scientific Orion Five Stars probe). These four variables are essential for describing the environmental dynamic and estuarine ecology and for depicting seasonal hydrologic changes and contaminants influence (Marshall & Elliott, 1998; Pombo et al., 2005; Rashed-Un-Nabi et al., 2011; Whitfield, 1999). Moreover, sediment samples were collected using a core sampler tube with a diameter of 50.8 mm; the first 15 cm of sediment were collected and divided into 5 cm thick layers (0–5 cm, 5–10 cm, and 10–15 cm). Additionally, the particle size, OM content, and THg were analyzed in each of the depth layers in subsamples of each collected core.

### Laboratory work

The OM content in sediment samples was determined through calcination at 450 °C for 4 h (Danovaro, 2010). The content of carbonates in the sediments of Buenaventura has not been reported, but since it is an estuary of sedimentary origin (Cantera & Blanco, 2001), the presence of carbonated biogenic material is low, so carbonates do not influence OM measurements. The granulometric characteristics of sediments were assessed through sieving (1 mm, 500 µm, 250 µm, 100 µm, 50 µm, and catch pan). The fractions retained in each sieve were classified using a modification of the Wentworth scale into three categories: coarse sediments (>500 µm), medium sediments (100–500 µm), and fine sediments (<100 µm) (Danovaro, 2010; Giménez et al., 2014; Silva & Astorga, 2010). In order to determine the THg (dry weight), sediment samples were lyophilized, pulverized, and homogenized, and then, 0.1–0.15 g of sample was analyzed using a direct mercury analyzer (DMA-80 Milestone), with a detection limit of 0.0001 µg g^−1^, following Method 7473 proposed by the EPA (US EPA, 1998). A calibration curve with R^2^ > 0.99 was obtained for all THg quantifications, using IAEA-405 certified reference material (0.0055 mg kg^−1^). Quality controls and reference material for the calibration curve were measured at least twice, making sure that variability between values for the same sample was <10% and that recovery percentages were at least 95%.

### Statistical analysis

The differences among hydroclimatic seasons were determined by a cluster analysis of the monthly rainfalls from 1995 to 2015 in the area of study. For this, a similarity matrix based on Euclidean distances (non-transformed data) was built, and the groups were identified using the mean group model (Clarke, 1993; Clarke & Warwick, 2001). For the grain size analysis, the sediments were grouped based on their size: coarse, medium, and fine. Coarse sediments included gravel and coarse sand (>500 µm); medium sediments comprised medium and fine sands (100–500 µm); and fine sediments comprised silt (coarse, medium, fine, very fine silt) and clay (<100 µm). The spatiotemporal changes and the differences in the sediment fractions regarding grain size, OM content, and THg were evaluated via permutational multivariate analysis of variance (PERMANOVA) of similarity matrixes based on Euclidean distances (non-transformed data) (Anderson, 2017; Clarke, 1993; Clarke & Warwick, 2001).

All the data of variables analyzed, including the THg data, were reviewed in search of extreme values using two standard deviations as a measure, to guarantee the quality of the data. The influence of grain size, OM content, and the physicochemical variables of the water column on the accumulation of mercury in sediments were evaluated through the generalized additive model (GAM) from Bayesian inferences. Traditional statistical models are often not suitable for representing complex systems with nonlinear relationship of variables (Rudy et al., 2016); however, GAM is a helpful tool for evaluating nonlinear complex relationships (Elith et al., 2008) and has been used in several studies (Amorós et al., 2018; de Souza et al., 2018; Tang et al., 2017). The model GAM with different combination of variables was evaluated using the Akaike information criterion (AIC) to compare and determine the more suitable model, considering ∆AIC < 2 (Krause et al., 2019; Martins et al., 2015).

## Results and discussion

### Results

#### Environmental variations of the water column

Temporal and spatial patterns were observed in the physicochemical variables of water (Fig. A2), including salinity, which presented higher values in the first semester (dry season 25.9 ± 0.6), and lower values in the second semester (rainy season 19.1 ± 0.8). Spatially, the lowest salinity (16.4 ± 0.9) was observed in the area closer to the mouth of the river (A1) in the rainy season, while the highest salinity (27.5 ± 0.7) was observed in the area with mainly marine influence (A4) in the dry season (Fig. A2a). Temperature presented minor variations between seasons; it was higher in the dry season (29.6 ± 0.2 °C) and lower in the rainy season (28.7 ± 0.1 °C). The highest temperature was detected in A4 (29.9 ± 0.6 °C) in the dry season, and the lowest was detected in A1 (28.3 ± 0.1 °C) in the rainy season (Fig. A2b). On the other hand, dissolved oxygen presented a temporal pattern, with higher concentrations (6.4 ± 0.2 mg l−1) in areas with predominant marine influence (A3 and A4) and lower concentrations (5.5 ± 0.1 mg l−1) in the interior areas (A1 and A2) (Fig. A2c). Furthermore, pH values showed small variations in the interior areas in both dry (A1: 7.8 ± 0.1; A2: 7.8 ±  <0.1) and rainy (A1: 7.7 ±  <0.1; A2: 7.8 ±  <0.1) seasons. However, pH seasonal variations were observed in the exterior areas (A3 and A4); A3 presented the main variability with the highest values (8.2 ± 0.1) in the dry season and the lowest values (7.7 ± 0.1) in the rainy season (Fig. A2d).

**Fig. 2:**
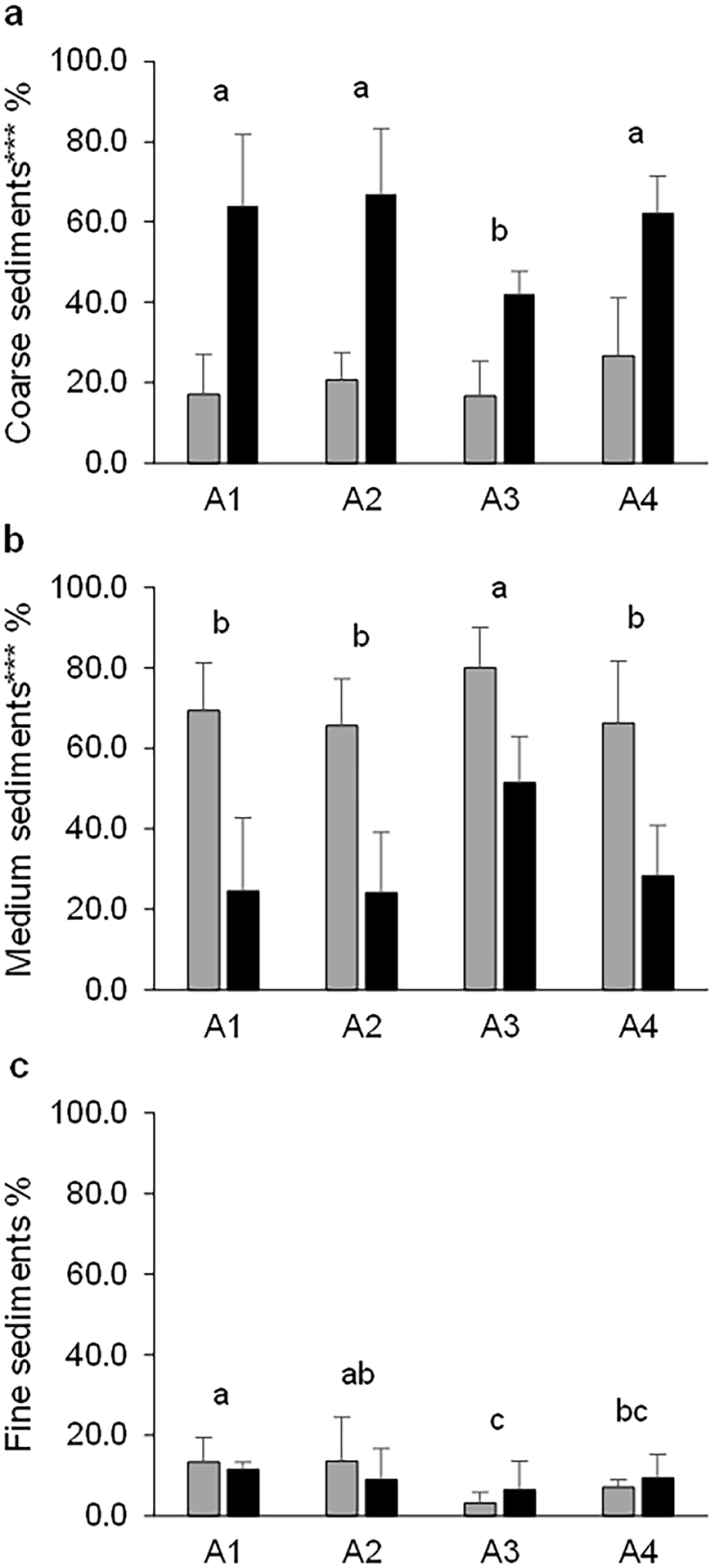
Mean ± SD of the different fractions of sediments based on their grain size. **a** Coarse, **b** medium, and **c** fine sediments based on seasons and areas of sampling. Dry season (grey bars) and rainy season (black bars). Area 1 (A1), area 2 (A2), area 3 (A3), and area 4 (A4). Significant differences between sampling areas are indicated with lower case letters within the figure (**a**, **b**, and **c**), above the bars. Areas with different letters are significantly different. Significant differences between sampling seasons are indicated with asterisks (p(PERM) < 0.001***)

#### Variations in grain size, OM content, and THg in sediments from different depths

In order to characterize the sediments, spatiotemporal and depth variations (sediment layers collected at 0–5 cm, 5–10 cm, and 10–15 cm depth) of the grain size, OM content, and THg were evaluated. The grain size (coarse sediments > 500 µm, medium sediments 100–500 µm and fine sediments <100 µm), OM content, and THg did not present significant differences (PERMANOVA) between depths or in the depth–area, depth–season, and depth–area–season interactions (p(PERM) > 0.05). However, significant differences were detected in the grain size between areas and season of sampling, and also in the area–season interaction (p(PERM) < 0.05). Similarly, OM content presented significant differences between areas, seasons, and in the area–season interaction (p(PERM) < 0.05). Significant differences in THg in sediments were found between sampling areas (p(PERM) = 0.0001) and sampling seasons (p(PERM) = 0.0001) but not in the area–season interactions (p(PERM) = 0.4487). Since no significant differences were detected in the grain size, OM content, or THg among the three deep sediment layers or their interactions, the spatiotemporal analysis of the grain size, OM content, and THg were assessed in the 0–5 cm layer, as it is in direct interaction with the water column.

#### Variations in grain size, OM content, and THg in superficial sediments

The coarse grain fraction of the superficial sediments (0–5 cm depth) showed significant differences between sampling seasons (p(PERM) < 0.001) with higher values in the rainy season (58.8% ± 15.9%) and lower values in the dry season (20.4% ± 10.5%). Significant differences were also detected between sampling areas (p(PERM) < 0.01): A1, A2, and A4 showed the highest values (40.5% ± 28.1%, 43.8% ± 26.8%, and 44.5% ± 21.9%, respectively) and A3 the lowest (29.4% ± 14.9%) (Fig. 2a). Medium sediments showed significant differences between sampling seasons (p(PERM) < 0.001) with higher values in the dry season (70.3% ± 13.0%) and lower values in the rainy season (32.1% ± 17.8%). At a spatial level, significant differences were detected between sampling areas (p(PERM) < 0.001), and contrary to that observed in the coarse grain fraction, medium sediments presented the highest values in A3 (65.7% ± 18.1%) and the lowest values in A1, A2, and A4 (47.0% ± 27.7%, 44.8% ± 25.2%, and 47.2 ± 23.9%, respectively) (Fig. 2b). Furthermore, fine sediments did not show significant differences between sampling seasons (p(PERM) > 0.05; dry season 9.4% ± 7.5% and rainy season 9.2% ± 5.9%). In contrast, significant differences were observed between sampling areas (p(PERM) < 0.05); the highest values were registered in A1 (12.5% ± 4.3%) and the lowest values in A3 (4.9% ± 5.3%) (Fig. 2c). The temporal differences may be related to changes in the amount of sediment entering the bay through the rivers, due to changes in precipitation.

**Fig. 3:**
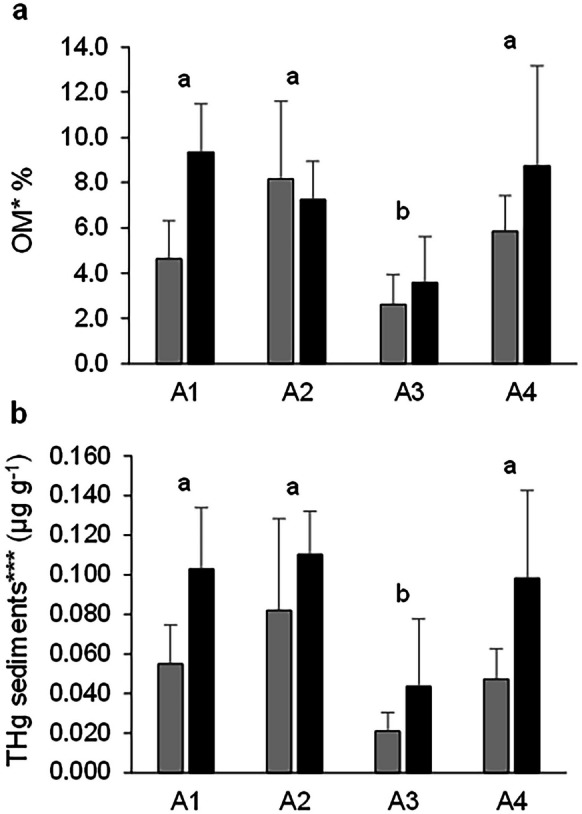
OM content (**a**) and THg in sediments (**b**), in function of seasons and sampling areas. Dry season (grey bars) and rainy season (black bars). Area 1 (A1), area 2 (A2), area 3 (A3), and area 4 (A4). Significant differences between sampling areas are indicated with lower case letters within the figure (**a** and **b**), above the bars. Areas with different letters are significantly different. Significant differences between sampling seasons are indicated with asterisks (p < 0.05*, p < 0.01**, and p < 0.001***)

The OM content of superficial sediments showed significant differences between season samplings (p(PERM) < 0.05) with a higher difference in the rainy season (7.2% ± 3.5%) and a lower difference in the dry season (5.3% ± 2.9%). Moreover, significant differences were observed between sampling areas (p(PERM) < 0.001); the highest values were found in A1, A2, and A4 (7.0% ± 3.1%, 7.7% ± 2.6%, and 7.3% ± 3.5%, respectively), and the lowest values were found in A3 (3.1% ± 1.7%) (Fig. 3a). Furthermore, THg in sediments presented values of 0.010–0.150 µg g^−1^, and significant differences were observed between seasons (p(PERM) < 0.001) with THg being higher in the rainy season (0.089 ± 0.041 µg g^−1^) and lower in the dry season (0.051 ± 0.033 µg g^−1^). Spatial significant differences (p(PERM) < 0.001) were found; the highest values were observed in A1, A2, and A4 (0.079 ± 0.035, 0.096 ± 0.038, and 0.073 ± 0.041 µg g^−1^, respectively), and the lowest values were observed in A3 (0.032 ± 0.027 µg g^−1^) (Fig. 3b). No significant differences were registered in the area–season interaction of OM content and THg in sediments.

#### Influence of grain size, OM content, and physicochemical variables of the water column on THg in sediments

In order to evaluate the influence of grain size, OM content, and the physicochemical variables of the water column on THg in sediments, all the variables were analyzed, individually and together. Variables associated with the physicochemical characteristics of the sediments showed a higher correlation with THg than those associated with the physicochemical characteristics of water (Table 1). The individual variables that significantly affected THg in sediments were OM content, coarse, medium, and fine sediments, salinity, dissolved oxygen, and pH. The variables related to the characteristics of sediments showed an explained deviation higher than 40% and that OM content (75.2%) and medium sediments (62.6%) showed the highest individual influence on THg in sediments.

**Table 1:** Univariate correlations between grain size, OM content, and physicochemical variables of water, and THg in sediments by GAM univariate analysis, where edf is the degree of the polynomial smooth function

	**edf**	**Explained deviation (%)**	**Adjusted R**^**2**^	**F-value**	***p-value***
Sediments					
Coarse sediments	1.00	41.00	0.40	31.94	**0.001**
Medium sediments	1.00	62.60	0.62	76.92	**0.001**
Fine sediments	2.06	49.20	0.47	16.33	**0.001**
OM content	1.47	75.20	0.74	73.81	**0.001**
Water physicochemical characteristics					
Salinity	3.17	24.4	0.19	2.86	**0.033**
Temperature	1.00	4.93	0.03	2.38	0.129
Dissolved oxygen	3.30	32.30	0.27	4.49	**0.003**
pH	2.79	22.80	0.18	2.89	**0.031**

The statistically significant variables are shown in bold

Regarding sediment related variables, although coarse sediments showed a direct linear correlation with THg, they also showed the lowest correlation with THg in sediments (Table 1 and Fig. A3a). Medium sediments showed a strong inverse linear correlation with THg in sediments (Table 1 and Fig. A3b). Furthermore, fine sediments presented a nonlinear correlation with THg in sediments; a diminution in accumulation was observed when the percentage of fine sediments was higher than 15% (Table 1 and Fig. A3c). The OM content showed a strong tendency toward a linear association with THg in sediments, and a direct correlation between these variables was found (Table 1 and Fig. A3d).

On the other hand, salinity presented a nonlinear relationship with THg with the highest THg values observed in the 16–19 range (Fig. A4a). Temperature showed an inverse linear relationship with THg and was the only variable with a nonsignificant correlation (Table 1 and Fig. A4b). Dissolved oxygen showed a nonlinear correlation with THg, and it was the physicochemical variable of water with the highest individual correlation. Moreover, the highest THg values were associated with concentrations of oxygen lower than 5.5 mg l^−1^ and higher than 7.5 mg l^−1^ (Table 1 and Fig. A4c). Furthermore, pH presented a nonlinear correlation with THg content; an increase in THg was observed for pH values lower than 7.8 (Table 1 and Fig. A4d). Due to the high number of individual correlations found, it was necessary to evaluate the influence of the environmental variables as a whole, to facilitate the understanding of the process.

**Fig. 4:**
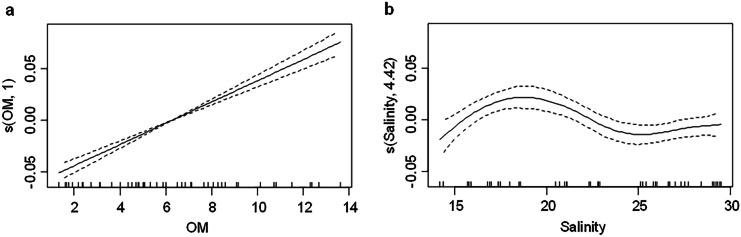
Multivariate GAM for THg in sediments according to the significant variables: **a** OM content and **b** salinity. The intermediate marks in the X-axis represent observed data. Y-axis shows THg in sediments in a fitted function. The number in the name of the y-axis represents the degree of the polynomial fitted by the model. The dotted lines show a range of two standard errors

The analysis of the variables together (grain size, OM content, and the physicochemical characteristics of the water column) that interacted and influenced THg in sediments (step GAM) indicated that the best model comprises OM content, coarse, medium, and fine sediments, salinity, temperature, and dissolved oxygen (Table 2). This model showed positive correlations with OM content and temperature; negative correlations with coarse and medium sediments and dissolved oxygen; and nonlinear correlations with fine sediments and salinity (Table 2). The model has a considerably high adjustment (R^2^ = 0.906; explained deviation = 92.7%), but only OM content and salinity were statistically significant. Therefore, we can assume that the adjustment of the model is overestimated due to the inclusion of non-significant variables (Table 2). Considering these results, a model that only includes the two significant variables was generated. These changes accounted for a model with high parsimony and good adjustment (R^2^ = 0.841; explained deviation = 85.9%; Table 2). In this model, OM content presented a positive linear correlation (Fig. 4a) and salinity presented a nonlinear correlation with the highest THg values detected in sediments with a salinity between 17 and 22 (Fig. 4b).

**Table 2:** Multivariate models to explain the influence of grain size, OM content, and the physicochemical variables of water on THg in sediments by step GAM analysis

	**Step GAM**	**Reduced Step GAM**
n	48	48
Adjusted R^2^	0.91	0.84
Explained Deviation (%)	92.70	85.90
AIC	−313.012	-
*Coefficient or degree of the polynomial*		
OM content	(0.010)***	(0.010)***
Coarse sediments	(−0.286)	-
Medium sediments	(−0.287)	-
Fine sediments	1.872	-
Salinity	3.533**	4.420**
Temperature	(0.002)	-
Dissolved oxygen	(−0.007)	-
pH	-	-

The “step GAM” column presents the general model and the “reduced step GAM” column presents the depurated model based on the significant variables

The numbers between brackets represent lineal correlations, and the asterisks indicate the statistically significant variables in the models

*p < 0.05; **p < 0.01; ***p < 0.001

## Discussion

As commonly described for a positive estuary, in this study, physicochemical variables, such as salinity, temperature, dissolved oxygen, and pH, showed variations associated with the different seasons and areas of the estuary (Day et al., 2012). The highest values for all the variables were registered in the dry season and in the outer part of the estuary, due to the lower rainfalls, the decrease in the entry of freshwater, and the spatial gradient (Barletta & Lima, 2019; Sun et al., 2009; Wolanski et al., 2004, 2006). The increase in salinity is related to not only a diminution in the entry of freshwater but also an increase in the marine influence, which creates the spatiotemporal environmental dynamics proper of an estuary (Day et al., 2012).

The higher temperatures registered in the dry season could be due to lesser cloudiness, as a higher incidence of solar radiation is observed in this season (Diaz, 2007; Otero, 2005). In addition, the highest temperature has been registered from April to June compared to the lower temperature generally observed from December to March, principally in years where there is not influence of the climatic phenomenon ENSO-El niño (Málikov & Villegas, 2005). At a spatial level, the higher temperature observed in the exterior area can be associated with the lower influence of the rivers providing water of a lower temperature, as these rivers originate in the Andes Mountains and are short and torrential (Cantera & Blanco, 2001; Diaz, 2007). On the other hand, the increment in the concentration of dissolved oxygen and pH could be related to an increase in the marine influence of the estuary during the dry season in the exterior areas. Accordingly, in tropical estuaries, higher concentrations of dissolved oxygen and pH are reportedly associated with a higher presence of marine conditions (Chango & Nacimba, 2015; Costa et al., 2018), which has also been observed in Buenaventura Bay (Duque et al., 2020; Gamboa-García et al., 2018; Molina et al., 2020).

The analysis of sediment layers obtained at different depths did not presented statistically significant differences regarding grain size, OM content, and THg. The homogeneity observed could be explained by the resuspension and mixing of sediments due to anthropomorphic interventions, such as dredging and construction of port infrastructures (Bertina et al., 2015; Chakraborty et al., 2019; Cukrov et al., 2020; van Maren et al., 2015) and the hydrodynamic variations associated with changes in the entry of freshwater, river runoff, and tides (Cantera & Blanco, 2001; Quintana et al., 2020). Moreover, in Buenaventura Bay, the sediment resuspension has been strongly associated with dredging, producing negative effects on water quality and artisanal fisheries (Montenegro & Torres, 2016; INVEMAR, 2017). No information is available for this estuary on sedimentation rates, which could be very useful for understanding sediment dynamics.

Regarding the sediment grain size, it showed a higher proportion of coarse and medium sediments and a lower proportion of fine sediments. The type of sediment was identified as sandy silt according to Shepard (1954). These differences could be related to the great amount of water delivered by the rivers in the estuary (1254 m^3^s^−1^) (Cantera & Blanco, 2001), in accordance with previous studies on Buenaventura Bay (Gamboa-García et al., 2018; Lucero et al., 2006). In this study, coarse and medium sediments showed inverse spatiotemporal values; the presence of coarse sediments was higher in the rainy season and in areas highly influenced by the rivers and the sea, while medium sediments were more abundant in the dry season and areas with intermediate river and ocean influence. This contrasting behavior may be explained by the hydrodynamic variations in the flow of the rivers, which increases the transport of coarse particles in the rainy season while favoring the transport of smaller particles in the dry season (Li & Li, 2018; Quintana et al., 2020). At a spatial level, the differences between coarse and medium fractions have been observed in different estuaries, possibly because of the hydrodynamic interaction between freshwater flows and marine currents (Garcia-Ordiales et al., 2018; Vane et al., 2020). The lower presence of fine sediments has been reported for Buenaventura Bay (Gamboa-García et al., 2018) and other estuaries (Vane et al., 2020; Garcia-Ordiales et al., 2018), and its spatial variability could be related to hydrodynamic differences that make the deposit of fine particles (Gamboa-García et al., 2018) and the resuspension of sediments produced by dredging operations (van Maren et al., 2015).

The content of OM and THg in superficial sediments were higher in the rainy season and at the extremes of the estuary and showed a correlated pattern. These correlations could be caused by the generation of OM–THg complexes, which are fundamental in the accumulation and mobility processes of mercury in sediments (Azaroff et al., 2019; Han et al., 2006; Lacerda & Fitzgerald, 2001). The higher OM content and THg in sediments during the rainy season may be related to an increase in the discharges of the rivers and runoffs, allowing the entry of dissolved OM and mercury, as it has been registered in Buenaventura Bay and other estuaries (Barletta et al., 2012; Gamboa-García et al., 2020; Gębka et al., 2020; Saniewska et al., 2018; Tamm et al., 2008). Furthermore, the high concentrations in the areas with river influence are probably caused by the river discharges, since they are a source of entry of heavy metals in estuaries (Cogua et al., 2012; Gamboa-García et al., 2020; Kehrig et al., 2003; Saniewska et al., 2018; Vane et al., 2020). In contrast, the high THg found closer to the ocean could be explained by a higher salinity and pH, which also favor mercury deposit in sediments (Cogua et al., 2012; Fiorentino et al., 2011; Saniewska et al., 2022; Vane et al., 2020). Moreover, fine sediments and the OM traveled longer towards the ocean due to the high river current, which finally mix at the outer part of the estuary (Chakraborty et al., 2019).

The mercury values found in this investigation (0.069 ± 0.042 µg g^−1^) were lower than those reported in another investigation for the mouth of the Dagua River (0.718 ± 0.736 µg g^−1^) in the Buenaventura Bay (Lucero-Rincón et al., 2023), which is one of the possible sources of mercury for the bay. Likewise, the concentration of mercury in the Buenaventura Bay is lower than that registered in estuaries in the south and southeast-eastern of Brazil (0.100 ± 0.050 µg g^−1^) (Trevizani et al., 2021), but higher (0.010–0.150 µg g^−1^) than that registered in the Polish part of the south of the Baltic Sea (0.003–0.077 µg g^−1^) (Jędruch et al., 2019). It is important to highlight that although the values of THg in sediments are relatively low, its effects on the ecosystem are evident, with active processes of THg accumulation in macroinvertebrates and fish being observed (Gamboa-García et al., 2020; Molina et al., 2020).

All the variables measured in sediment and in the water column were individually correlated with THg in sediments, except for temperature, which suggests a complex dynamic of the distribution and accumulation processes of mercury in sediments in Buenaventura Bay (Chakraborty et al., 2019; Quintana et al., 2020). This complexity has been associated with the environmental dynamic proper of the estuarine ecosystems (Elliott & Quintino et al., 2007; Teichert et al., 2017; Li & Li, 2018; Pérez-Ruzafa et al., 2019). This variation in mercury distribution and accumulation could be produced by the interaction of different environmental variables with the chemical and hydrodynamic processes controlling mercury deposit and resuspension (Chakraborty et al., 2019; Curtis et al., 2019; Fiorentino et al., 2011; Garcia-Ordiales et al., 2018; He et al., 2019; Padalkar et al., 2019; Wasserman et al., 2002).

Individual correlations among THg and the type of sediments showed opposite patterns for spatiotemporal abundance. The direct correlation between coarse sediments and THg could be due to a higher number of available particles of large size, favoring mercury binding and accumulation (Padalkar et al., 2019). This correlation depends on the fraction of sediments binding to the OM (He et al., 2019; Padalkar et al., 2019), where, OM content showed the highest values in the same seasons and areas of sampling than coarse sediments. In contrast, there was an inverse correlation between medium sediments and THg, and fine sediments and OM content presented an individual nonlinear correlation with THg. This positive correlation could be explained by the association of OM with fine sediments and the strong binding between mercury and OM because of their chemical affinity that facilitates the adsorption processes (Cukrov et al., 2020; Vane et al., 2020; Zhao et al., 2019). This correlation has been observed in Buenaventura Bay (Gamboa-García et al., 2020) and in other estuaries (Garcia-Ordiales et al., 2018; Quintana et al., 2020).

The individual influence of the physicochemical variables of water on THg in sediments was nonlinear. Salinity and pH represented the temporal variations as they showed the highest THg in sediments in freshwater conditions, while dissolved oxygen represented the spatial variations, with the highest THg in sediments in areas predominantly influenced by the rivers (low dissolved oxygen) and sea (high dissolved oxygen). Furthermore, the highest THg in sediments observed in conditions of low salinity may correlate to the entry of mercury through rivers and runoff (Chen et al., 2020; Li et al., 2020; Saniewska et al., 2018) and to a reduction in mercury competition in low salinity conditions wherein the metal stays bound to the OM, remaining fixed in the sediments and increasing its concentration (Fiorentino et al., 2011; Wasserman et al., 2002). Thus, the results of this study agree with those of other estuaries (Cogua et al., 2012; Gamboa-García et al., 2020; Kehrig et al., 2003). Moreover, as has been reported, influence of salinity on THg in sediments can vary according to the intrinsic characteristics of each system (Curtis et al., 2019; Garcia-Ordiales et al., 2018; Li et al., 2020).

The highest THg values in sediments were found at pH 7 and 7.8, probably because these conditions favor the generation of metal–sulfur complexes (Rickard & Luther, 2007) that facilitate mercury accumulation (Quintana et al., 2020). In this study, this pH range can be associated with the influence of freshwater in the estuary, which could be related to the entry of mercury through the river’s discharges (Saniewska et al., 2018). The high THg values observed in sediments with low dissolved oxygen could be explained by its association with areas of river influence, which are important entry points of mercury to the estuaries (Saniewska et al., 2018). Furthermore, low pH values may be associated with OM decomposition (Day et al., 2012), which is related to THg in sediments (Azaroff et al., 2019; Han et al., 2006; Lacerda & Fitzgerald, 2001). Moreover, high THg values in sediments related to high content of dissolved oxygen, associated in this study with marine conditions, may correspond to physicochemical variations induced by oxygen, such as changes in the redox potential (Quintana et al., 2020), and hydrodynamic variations, such as deceleration of water flows (Chakraborty et al., 2019), that facilitate mercury accumulation in sediments.

The individual influence of multiple variables on the distribution and accumulation dynamics of mercury in sediments, suggest an evaluation of the synergistic effects and the reduction of interactions to better understand the distribution and accumulation processes of mercury. However, in this study, only two variables (salinity and OM content) were significant when the model was simplified, as recommended in studies on mercury and other contaminants (Foster et al., 2019; Kim et al., 2020; Thomas et al., 2020). In the simplified model, OM content may constitute the mean of transport and accumulation of mercury, while changes in salinity could account for the spatiotemporal gradients of the hydroclimatic and environmental dynamic and its effects on the distribution and accumulation processes of mercury in the estuarine sediments. The inclusion of OM in the model could be due to its chemical characteristics as OM facilitates mercury absorption and the generation of OM–Hg complexes, which are important factors for the mobility and accumulation of mercury in estuaries (Lacerda & Fitzgerald, 2001; Han et al., 2006; Azaroff et al., 2019; Padalkar et al., 2019; Zhao et al., 2019; Cukrov et al., 2020; Vane et al., 2020). Moreover, salinity is the best descriptor of the spatiotemporal dynamics in estuaries (Barletta & Lima, 2019; Day et al., 2012; Whitfield et al., 2012), and changes in this parameter reflect the variations in the entry of freshwater, which is an important source of mercury (Chen et al., 2020; Li et al., 2020; Saniewska et al., 2018), as has been reported for Buenaventura Bay (Gamboa-García et al., 2020).

## Conclusions

The variations in the spatiotemporal variables analyzed in this study represent an environmental gradient within estuarine conditions. Sediments did not presented significant differences between the layers obtained at different depths, or in grain size, OM and THg. In superficial sediments, OM and THg showed similar variation patterns, which were higher in the rainy season and the interior part of the estuary. Furthermore, the individual influence of multiple variables shows a complex dynamic of the distribution and accumulation processes of mercury in sediments of Buenaventura Bay. The model that better explained THg accumulation in sediments was based on OM and salinity. The OM presented a proportional direct relationship with THg and possibly represented the main pathway of transport and accumulation of mercury. The salinity showed a nonlinear relationship with THg, where the highest THg value was found between 17 and 22. The changes in salinity represent the influence of the hydroclimatic variations and environmental gradients of the estuary.

### Supplementary Information

Below is the link to the electronic supplementary material.

Supplementary file1 (PDF 238 KB)

## Data Availability

The data in this study are available upon reasonable request to the corresponding author.
