# Supplementary material for: Effect of environmental variables on mercury accumulation in sediments of an anthropogenically impacted tropical estuary (Buenaventura Bay, Colombian Pacific)
Source: Environ Monit Assess. 2023 Oct 14;195(11):1316. doi: 10.1007/s10661-023-11721-9 (PMC10575815; doi:10.1007/s10661-023-11721-9)
Supplement: Supplementary file 1 — Supplementary file1 (PDF 238 KB) [file 10661_2023_11721_MOESM1_ESM.pdf]

Appendices

Seasons according to rainfall

The cluster analysis of the monthly rainfalls registered between 1995 and 2015 divided the months of the year into three groups (Fig. A1a): group I includes the months of the rainy season (July, September, October, November, and December), with rainfalls above 500 mm; group II comprises the months of the dry season (January, February, March, April, and June), with rainfalls lower that 500 mm; and group III includes the months with variable rainfalls (May and August). However, considering the magnitude of rainfalls and calendar location of the months of group III, May and August were classified as months of the dry and rainy season, respectively (Fig. A1b); therefore, two sampling groups were finally analyzed.

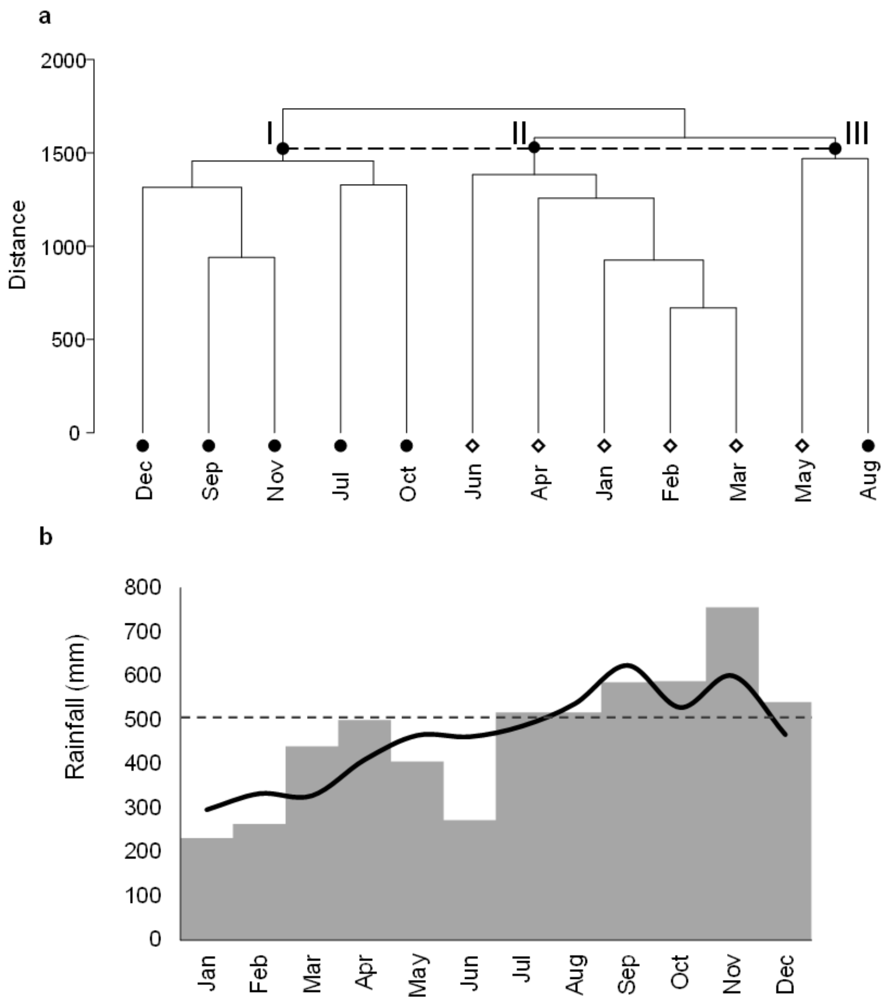

Fig. A1 Rainfalls in the Buenaventura Bay. (a) Total monthly rainfall in 2015 (grey bars) and historic rainfall pattern (1931–2015) (black line). (b) Cluster analysis of rainfalls. The data was provided by the Institute of Hydrology, Meteorology and Studies, IDEAM for its acronym in Spanish (http://www.ideam.gov.co/).

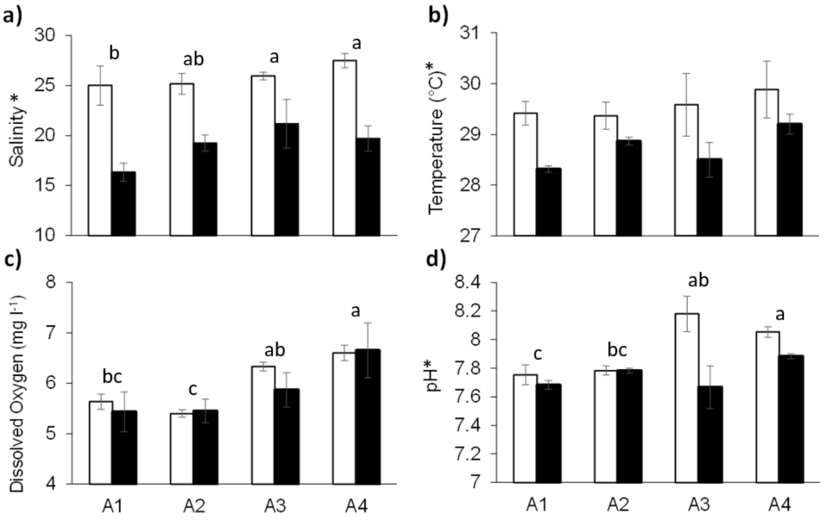

Fig. A2 Environmental variables: (a) salinity, (b) temperature, (c) dissolved oxygen, and (d) pH (mean ± SD) in function of the seasons and sampling areas. Rainfall < 500 mm in the dry season (white bars), and Rainfall > 500 mm in the rainy season (black bars). Area 1 (A1), area 2 (A2), area 3 (A3), and area 4 (A4). Significant differences between sampling areas are indicated with lower case letters within the figure (a, b, and c), above the bars. Areas with different letters are significantly different. Significant differences between sampling seasons are indicated with asterisks (p(PERM) < 0.05*).

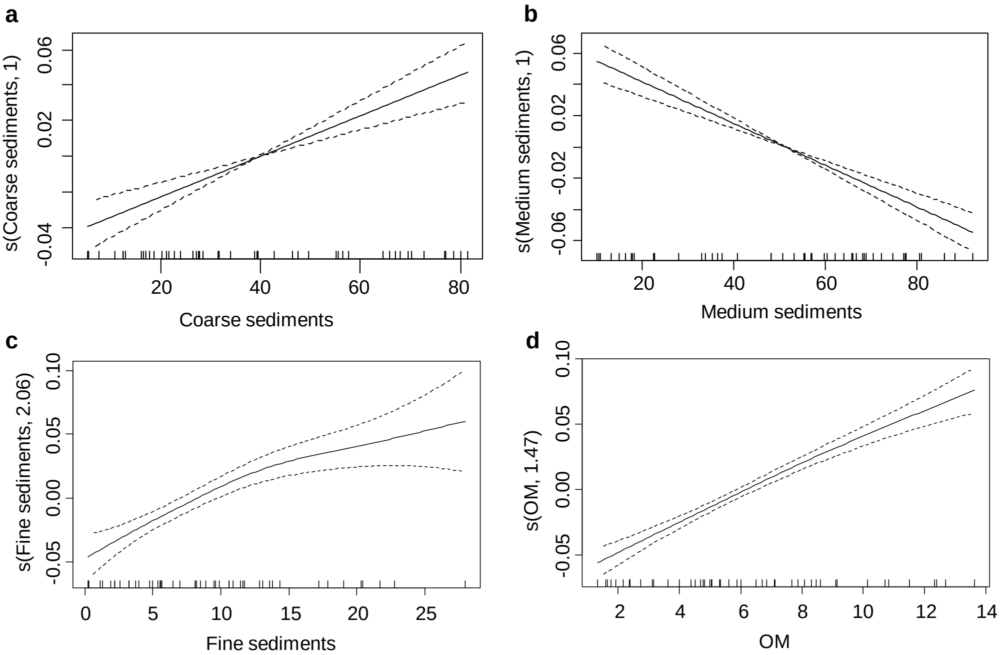

Fig. A3 Individual GAMs of THg in sediments as a dependent variable, and grain sizes and OM content as independent variables. The intermediate marks in the X-axis represent observed data. Y-axis shows THg in sediments in a fitted function. The number in the name of the y-axis represents the degree of the polynomial fitted by the model. The dotted lines show a range of two standard errors

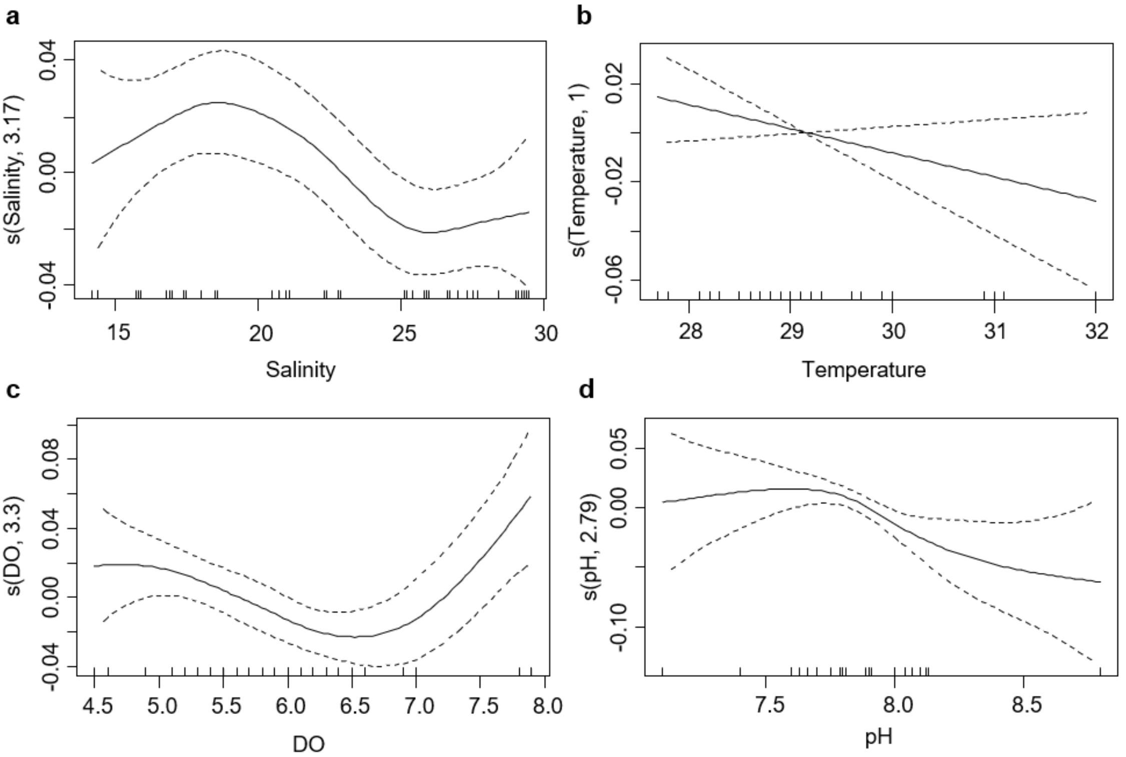

Fig. A4 Individual GAMs of THg in sediments as a dependent variable, and the physicochemical variables of the water column as independent variables. The intermediate marks in the X-axis represent observed data. Y-axis shows THg in sediments in a fitted function. The dotted lines show a range of two standard errors
